# Friend or Enemy: A Dual Role of Autophagy in Plant Virus Infection

**DOI:** 10.3389/fmicb.2020.00736

**Published:** 2020-04-21

**Authors:** Xiuqin Huang, Siping Chen, Xiaorong Yang, Xin Yang, Tong Zhang, Guohui Zhou

**Affiliations:** ^1^Guangdong Province Key Laboratory of Microbial Signals and Disease Control, College of Agriculture, South China Agricultural University, Guangzhou, China; ^2^Guangdong Laboratory of Lingnan Modern Agriculture, South China Agricultural University, Guangzhou, China

**Keywords:** plant virus, autophagy, ATGs, antiviral mechanism, counter-defense

## Abstract

Autophagy is a primary protective process that involves removing damaged organelles or dysfunctional proteins in eukaryotes. The autophagy pathway not only maintains cellular homeostasis, but also modulates the host's cellular response to pathogen infection. Several studies proved that autophagy plays a dominant role in plant fitness and immunity. As intracellular parasites, the replication and spread of viruses entirely rely upon the molecular machinery of the host cell, including the autophagy process. Plant viruses severely affect crop yields and quality. During infection, complex interactions occur between viral proteins and host factors in relation to plant defense and virus counter-defense. An increasing number of studies demonstrated that plants use autophagy to eliminate and inhibit viruses; some viruses were shown to manipulate the process of autophagy to promote their own replication and survival in plant cells. In this review, we summarize recent advances in plant autophagy, with an emphasis on the role of autophagy in plant virus infection.

## Introduction

Autophagy is a conserved intracellular degradation pathway through which damaged organelles, non-functional proteins, and harmful microbial invaders are delivered to vacuoles in yeast and plants or lysosomes in animals to be degraded (Liu and Bassham, 2012; Marshall and Vierstra, 2018; Shimamura et al., 2019). This process achieves intracellular recycling and plays a paramount role in energy balance. The autophagy phenomenon was first observed in 1963, but its mechanism was not revealed until 1993 (Deter and de Duve, 1967; Tsukada and Ohsumi, 1993). The mechanism of autophagy in yeast was characterized first, with researchers making considerable progress in plant models for more than a decade. The genes involved in autophagy were named autophagy-related genes (*ATG*s) (Klionsky et al., 2003; Mizushima et al., 2011). In recent years, many *ATGs* have been identified in *Arabidopsis*, tobacco, rice, and many other plants (Yoshimoto et al., 2010; Xia et al., 2011; Yoshimoto, 2012; Zhou et al., 2015).

Plant viruses include some of the most devastating crop pathogens, leading to significant agricultural losses worldwide and seriously threatening global food security (Oerke and Dehne, 2004; Fargette et al., 2006). Due to the systematic infection characteristics of plant viruses, no chemical pesticides target viral diseases directly. Autophagy is a major homoeostatic process through which cytoplasmic components are delivered to vacuoles as a regulatory pathway to coordinate the host's response to various intracellular pathogens, including viruses (Shoji-Kawata and Levine, 2009). Therefore, this pathway is a potential target for modulation by chemical agents or molecular breeding to establish resistance to viruses in crops.

**Figure 1:**
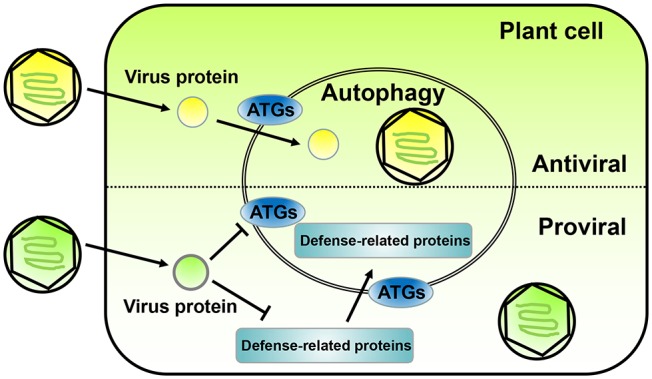
The opposite roles of autophagy in plant-virus interaction. The diagram on the top shows an antiviral role for autophagy during plant infection by viruses. Plant viruses express proteins (yellow ball) to promote infection; however, sometimes the autophagy of the plant infected cell is activated and directly or indirectly targets the virus protein or vision toward degradation. The diagram on the bottom shows a proviral role for autophagy during plant infection by viruses. In some cases, plant virus proteins (green ball) can interact with ATGs to inhibit autophagy and prevent degradation. Also, some plant virus encoded proteins can promote the degradation of plant defense-related proteins through the autophagy pathway. Both case scenarios lead to an enhancement of viral replication and infection in the host plant.

In the past decade, a lot of progress has been made in the relationship between plant autophagy and virus infection. Autophagy can promote virus infection or inhibit it (Figure 1). In this work, we summarize the current state of autophagy in plant systems and discuss the dual roles of autophagy in the arms race between the defense and counter-defense of plants and viruses, respectively.

## Autophagy and its Molecular Mechanism in Plants

Autophagy is an evolutionarily conserved process observed in yeast, plants, and animals, and whose regulatory mechanisms and induction factors are quite similar (Hurley and Young, 2017). The three major types of autophagy include macroautophagy, microautophagy, and molecular chaperone-mediated autophagy (CMA) (Mizushima et al., 2008; Kaushik and Cuervo, 2018). Macroautophagy (hereafter called autophagy) occurs when cytoplasmic components are engulfed by double-membrane structures, termed autophagosomes, and subsequently delivered to vacuoles for breakdown and turnover in plants (Ohsumi, 2001; Klionsky and Codogno, 2013; Marshall and Vierstra, 2018). Tremendous progress has been made regarding the molecular mechanism of macroautophagy. From its inception at the preautophagosomal structure (PAS), the phagophore expands into a cup-shaped structure and ultimately forms a double-membrane vesicle called an autophagosome (Marshall and Vierstra, 2018). By contrast, microautophagy refers to the direct depression of a plant's vacuolar membrane, with the trapped tonoplast swallowing cytoplasmic material into the vacuole for degradation. However, information regarding microautophagy is limited and no reliable markers are available for monitoring purposes (Li et al., 2012). CMA targets proteins bearing the KEFRQ residue, which is recognized by the chaperone heat shock-cognate protein that is 70 KDa in mass (Hsc70). The chaperone delivers the substrate to the lysosome where they bind to lysosome-associated membrane protein type 2A (LAMP-2A), allowing the soluble proteins to be selectively degrade. To date, the CMA pathway has been described only in mammals and birds (Cuervo and Wong, 2014; Catarino et al., 2017; Kaushik and Cuervo, 2018). The autophagy process of plants includes the initiation of autophagy, nucleation, elongation, completion, fusion with vacuoles, and breakdown. These are also the key steps in the life-cycle of an autophagosome (Lamb et al., 2013; Ismayil et al., 2019).

**Figure 2:**
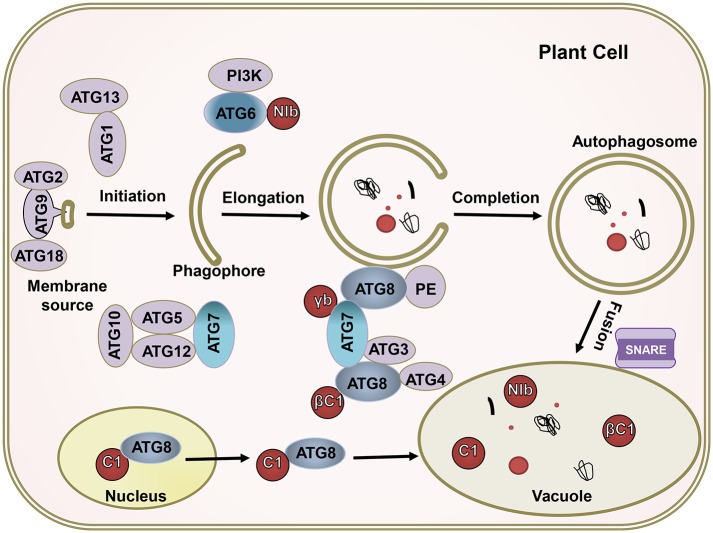
The ATGs target viral proteins for degradation by the autophagy pathway. The autophagy pathway includes four key steps including initiation, elongation, completion, and fusion. Most ATGs drive this process; βC1 of geminivirus, NIb of PVY, γb of BSMV, and C1 of TLCYnV; these virus effectors (red) interact with crucial autophagy genes, like ATG6, ATG7, and ATG8. The ATGs target viral proteins for their degradation.

The complicated molecular machinery of autophagy has been unveiled over the past 15 years (Klionsky and Codogno, 2013; Medina-Puche and Lozano-Duran, 2019; Signorelli et al., 2019). Autophagy, which always occurs at a basal level in all plant cells, is responsible for the elimination of harmful cellular debris (Marshall and Vierstra, 2018). The execution of autophagy requires many ATG proteins (Wang et al., 2018; Levine and Kroemer, 2019). When cells are stimulated by nutrient-starved conditions, abiotic stresses, or pathogen infection, the activity of TOR kinase in plant cells is inhibited and ATG13 is rapidly dephosphorylated. The dephosphorylated ATG13 then binds to ATG1 to form an activated ATG1 kinase complex called an autophagy precursor, thereby initiating autophagy as a key activator. Autophagy precursors interact with PI3K kinase complexes (including ATG6/Vps30, ATG14, etc.) to form autophagic vesicles, in which the ATG9 circulation system (including ATG2, ATG9, ATG18, etc.) is involved in forming the membrane of the autophagic vesicles. Under the action of two ubiquitin-like systems, i.e., the ATG8 lipidation system (including ATG3, ATG4, ATG7, and ATG8) and the ATG12 conjugation system (including ATG5, AT7, ATG10, ATG12, and ATG16), the autophagosomes gradually mature (Xie and Klionsky, 2007; Yoshimoto et al., 2010). Finally, the soluble N-ethylmaleimide-sensitive factor attachment protein receptor (SNARE) mediates the fusion of the autophagosome and vacuole, thereby forming an autolysosome and causing the autophagosomal content to bedegrade in the vacuole (Wang et al., 2016) (Figure 2).

## Methods for Assessing Autophagy During Plant Virus Infection

The establishment of suitable approaches to assess autophagy is a key challenge in revealing the role of the pathway in plant and virus interaction. Here, we describe some common methods of autophagy investigation.

### Transmission Electron Microscope Observation

Direct observation of autophagosomes by transmission electron microscope (TEM) is an effective method of analyzing autophagy (Klionsky et al., 2016). TEM allows for the visualization of the autophagosome's double-membrane circular structure, which has a diameter from 100 to 1000 nm in plant cells; the electron density of the contents in an autophagosome is similar to that of cytoplasm. Exhibiting the morphology of autophagosomes by TEM in nanometer range can help researchers confirm the occurrence and dynamics of autophagy during virus infection (Barth et al., 2010; Klionsky et al., 2016; Yoshii and Mizushima, 2017).

### Fluorescent Protein-Based Autophagy Monitoring

The ATG8 lipidation system is essential for autophagosome formation. Therefore, fluorescent proteins (FPs) that are fused with ATG8 under laser scanning confocal microscope (LSCM) can be used to track autophagy in plant cells. ATG8 is located on both the inner and outer membranes of autophagosomes. After an autophagosome fuses with a vacuole, the autophagic bodies in the vacuole also exhibit the ATG8 protein (Bassham, 2015). Compared with other detection methods, FP-ATG8 fusion protein accurately quantifies autophagosomes in living cells (Bassham, 2015; Izumi et al., 2015). Many plant species encode several ATG8 homologs, all of which are located on autophagosomes and allowing most of them to be used as markers of autophagic vesicles (Bassham, 2015). FP-ATG8 fusion protein can be expressed consistently in transgenic plants or transiently by agrobacterium inoculation, allowing autophagy in living plant cells to be monitored in real time (Contento et al., 2005; Bassham, 2015; Izumi et al., 2015).

### Methods for Assessing Autophagic Flux

Autophagic flux refers to the entire process of autophagy. The most common method to indicate autophagic flux in plants is by detecting the protein level of ATG8-PE or NBR1 by Western blot, which provides reliable evidence about autophagic flux (Minina et al., 2013; Klionsky et al., 2016). ATG8 is covalently attached to the lipid phosphatidylethanolamine (PE) to produce ATG8–PE, which is imbedded in the membrane of the autophgosome. Thus, ATG8 and ATG8–PE turnover indicates the strength of autophagic flux. NBR1, as an autophagy substrate, can be degraded by autophagy pathway; the protein level of NBR1 indicates autophagy activity. In addition, assays using pharmacological drugs affecting vacuolar hydrolases are quite similar to those performed in mammalian cells with drugs affecting lysosomal hydrolases, which will benefit the observation of autophagic flux. Concanamycin A (ConA), an inhibitor of vacuolar H^+^-ATPase, can block vacuolar degradation, while E64d, an inhibitor of lysosomal/vacuolar cysteine proteases, can also block vacuolar degradation. Autophagic bodies within the vacuole are in proportion to autophagic activity. The autophagic bodies can be observed by fluorescence microscopy when labeled with fluorescent protein fused ATG8, like GFP-ATG8, or can be monitored by TEM (Bassham, 2015; Li et al., 2018).

### Chemical Inducers and Inhibitors of Autophagy

Besides the above cell biological means used to directly observe autophagy, various chemical inhibitors affect different stages of autophagy which can be used to test the role of autophagy in virus infection. Rapamycin acts as an allosteric inhibitor of mTOR upon association with the FK506-binding protein 12 (FKBP12) (Yang et al., 2013); however, many plants are insensitive to it (Xiong and Sheen, 2015). Another TOR inhibitor, AZD8055, an efficient autophagy inducer that works by suppressing TOR signaling, is widely used in plants (Dong et al., 2015; Pu et al., 2017; Song et al., 2019). Several inhibitors of autophagy in plant cells have been reported to impact on different stages of the process. ConA blocks vacuolar hydrolysis and suppresses autophagic vesicle degradation in vacuoles (Hafrén et al., 2017), whereas 3-methyladenine (3-MA) inhibits PI3K activity, and E64d, a cysteine protease inhibitor, blocks the flux of autophagy (Takatsuka et al., 2004; Kim et al., 2008; Pasquier, 2016; Xu et al., 2017). These chemical inducers and inhibitors can be directly sprayed onto or inoculated into the plant leaf to trigger autophagy, thereby allowing the role of autophagy in relation to biotic or abiotic stresses to be investigated.

### Silencing or Mutation of *ATGs* in Plants

In addition to chemical treatments, numerous key *ATG* genes of plants are appropriate targets for autophagy inhibition. Virus-induced gene silencing (VIGS) is widely used to silence *ATG* genes to address whether autophagy plays a role in pathogen infection (Liu et al., 2005). In *Arabidopsis*, many *atg* T-DNA insertion mutants are available (Zheng et al., 2006; Yoshimoto et al., 2009; Zhou et al., 2013). For instance, *atg5* and *atg7* mutants were inoculated with cauliflower mosaic virus (CaMV) and exhibited more severe symptoms than the wild type, indicating that the process of autophagy may involve an antiviral mechanism (Hafrén et al., 2017). In recent years, the clustered regularly interspaced short palindromic repeats (CRISPR)/CRISPR-associated9 (Cas9) (CRISPR/Cas9) system was developed for genome editing (Doudna and Charpentier, 2014; Ma et al., 2016), allowing the scientific community to obtain *atg* mutants in different species and therefore promoting the study of the function and mechanism of autophagy (O'Prey et al., 2017; Norizuki et al., 2019).

## Plant Defense Against Virus Infection by Autophagy

Although autophagy provides different contributions to plant immunity, research on the relationship between plant autophagy and virus infection is relatively lagging. Liu et al. (2005) reported the first case of plant virus-induced autophagy using the fluorescent dye LysoTracker Red to probe autolysosomes in tobacco cells that were infected by tobacco mosaic virus (TMV). The mRNA and protein levels of Beclin1 increased in the early stages of the TMV-induced hypersensitive response (HR) of programmed cell death (PCD) in N gene-containing *Nicotiana benthamiana* plants. Knocking down the expression levels of *ATG3, Beclin1*, and *ATG7* using VIGS exhibited unrestricted HR PCD and demonstrated enhanced TMV accumulation (Liu et al., 2005). These results indicated that autophagy plays an essential role in the N-mediated defense against TMV and requires Beclin1 for induction. Recently, both PCD and reactive oxygen species (ROS) level were reported to be regulated by autophagy during the compatible interplay between plant and virus (Ismayil et al., 2019). Some other components, such as sugar levels, are also modulated by virus infection (Llave, 2016; de Haro et al., 2019). Whether sugar has a relationship with autophagy induction during plant virus infection remains to be investigated.

For most plant viruses, autophagy is an antiviral mechanism, which directly targets viral components for degradation (Figure 1, upper part and Table 1). Cotton leaf curl Multan virus (CLCuMV) induces autophagy in host plants, and the virulence factor βC1 of CLCuMV directly interacts with ATG8f, which is a key autophagy protein. This interaction occurs in autophagy vesicles and targets βC1 for breakdown. When *ATG5* or *ATG7* were silenced to inhibit the autophagy pathway, more severe symptoms were caused by the virus in plants, indicating that autophagy plays an important antiviral role in CLCuMV infections (Haxim et al., 2017). These same autophagy effects were observed in other geminiviruses, like tomato yellow leaf curl virus (TYLCV) and tomato yellow leaf curl China virus (TYLCCNV). Silencing of *ATG5* or *ATG7* caused more severe symptoms and increased viral DNA accumulation (Haxim et al., 2017), indicating that autophagy may be a general antiviral mechanism against diverse geminiviruses. Recently, a study clarified the mechanism of CLCuMV-encoded βC1 inducing autophagy (Ismayil et al., 2020). Cytosolic glyceraldehyde-3-phosphate dehydrogenase (GAPC), is a negative autophagy regulator that interacts with ATG3 to inhibit autophagy in *N. benthamiana* (Han et al., 2015). Wherever, βC1 interacts with GAPC by disrupting GAPCs-ATG3 interactions to activate autophagy pathway (Ismayil et al., 2020). Turnip mosaic virus (TuMV) of the potyvirus group also induces autophagy in plants. Beclin1 (ATG6) is a key protein that interacts with ATG8a to mediate the autophagy process; expression levels of Beclin1 were shown to be significantly upregulated by TuMV infection. Interestingly, Beclin1 directly interacts with NIb, the RNA-dependent RNA polymerase (RdRp) of TuMV, and targets NIb degradation via the autophagy pathway. Silencing *Beclin1* or *ATG8a* increased NIb accumulation and promoted TuMV infection (Li et al., 2018). Two other RNA viruses, namely cucumber green mottle mosaic virus (CGMMV) of the *Tobamovirus* genus and pepino mosaic virus (PepMV) of the *Potexvirus* genus, face similar antiviral mechanisms. Their encoded RdRps also interact with Beclin1, and silencing *Beclin1* caused more severe symptoms than those observed in control plants. As a critical autophagy regulator, Beclin1 restricts RNA virus infection via the autophagy pathway to suppress and degrade viral RdRps (Li et al., 2018). CaMV of the *Caulimovirus* genus also induces the formation of autophagosomes in *N. benthamiana* cells. In *Arabidopsis atg*-mutants, the symptoms caused by CaMV were more severe than in wild type plants. Intriguingly, CaMV-encoded P4 interacts with NEIGHBOR of BRCA1 (NBR1), which is an autophagy receptor selectively targeting polyubiquitinated aggregates to nascent autophagosomes (Kirkin et al., 2009; Svenning et al., 2011; Hafrén et al., 2017). The P4–NBR1–ATG8 complex promotes selective autophagy to target P4 and viral particles for degradation to fight against CaMV infection (Hafrén et al., 2017). Similarly, the autophagy cargo receptor NBR1 mediates selective autophagy by targeting helper-component proteinase (HC-Pro) and RNA-silencing suppressors (RSS) of TuMV for degradation to suppress viral accumulation (Hafrén et al., 2018). A study reported that the replication-initiator protein C1 of tomato leaf curl Yunnan virus (TLCYnV) induced plant autophagy. The direct interaction between C1 of TLCYnV and ATG8h led to the translocation of C1 from the nucleus to the cytoplasm, resulting in degradation by autophagy. This process depends on the interaction between ATG8h and XPO1a, which is a crucial component of the nuclear export pathway. Treatment with autophagy inhibitors or silencing of *ATG5, ATG7*, and *ATG8h* promoted TLCYnV infection in solanaceous plants (Li et al., 2019). These studies confirmed that the autophagy pathway of plants plays a direct antiviral role by interacting with virus-encoded proteins. In addition, the expression of TLCYnV-encoded C1 alone is sufficient to induce autophagy, although how TLCYnV or C1 protein activate autophagy is unclear (Li et al., 2019).

**Table 1:** Summary of reported interactions between plant virus and host factors involved in autophagy.

**Role**	**Virus**	**Host plants**	**Virus effector**	**Host factors**	**References**
Antiviral function	Cucumber mosaic virus	Tobacco	2b	rgs-CaM	Nakahara et al., 2012
	Tomato aspermy virus		2b	rgs-CaM	
	Turnip mosaic virus	Tobacco	HC-Pro	rgs-CaM	Nakahara et al., 2012
	Tobacco etch virus		HC-Pro	rgs-CaM	
	Clover yellow vein virus		HC-Pro	rgs-CaM	
	Turnip mosaic virus	Arabidopsis Tobacco	HC-Pro	NBR1	Hafrén et al., 2018
	Watermelon mosaic virus	Arabidopsis	HC-Pro	NBR1	
	Cauliflower mosaic virus	Arabidopsis Tobacco	P4	NBR1	Hafrén et al., 2017
	Cotton leaf curl Multan virus	Tobacco	βC1	ATG8	Haxim et al., 2017
	Tomato yellow leaf curl virus		βC1	ATG8	
	Tomato yellow leaf curl China virus		βC1	ATG8	
	Turnip mosaic virus	Arabidopsis Tobacco	NIb	Beclin1(ATG6)	Li et al., 2018
	Cucumber green mottle virus	Tobacco	RdRp	Beclin1(ATG6)	
	Pepino mosaic virus		RdRp	Beclin1(ATG6)	
	Tomato leaf curl Yunnan virus	Tobacco	C1	ATG8h	Li et al., 2019
Pro-viral function	Potato leafroll virus	Potato Arabidopsis	P0	AGO1	Derrien et al., 2012
	Turnip mosaic virus	Tobacco Arabidopsis	VPg	SGS3	Cheng and Wang, 2017
	Tobacco etch virus		VPg	SGS3	
	Soybean mosaic virus		VPg	SGS3	
	Rice stripe virus	Tobacco Rice	NSvc4	NbREM1/OsREM1.4	Fu et al., 2018
	Barley stripe mosaic virus	Tobacco Barley	γb	ATG7	Yang et al., 2018
	Cauliflower mosaic virus	Arabidopsis Tobacco	P6	NBR1	Hafrén et al., 2017

Besides the direct antiviral mechanism, autophagy is indirectly involved in plant defense against virus infection by targeting host factors (Table 1). Two well-studied RSSs, 2b and HC-Pro, of *Cucumovirus* and *Potyvirus* genera, were reported to interact with the regulator of gene silencing calmodulin-like protein (rgs-CaM), a cellular suppressor of post-transcriptional gene silencing (PTGS) in plants. The protein levels of endogenous rgs-CaM and viral RSSs significantly increased after autophagy inhibitor 3-MA treatment, indicating that rgs-CaM and RSSs were degraded by autophagy (Nakahara et al., 2012).

## Plant Virus Counter-Defense Acting on Autophagy

Plants defend against virus infection by autophagy, but, as a result of long-term co-evolution, viruses produced counter-defense strategies. As mentioned above, CaMV-encoded P4 interacts with NBR1 and promotes selective autophagy to target P4 and viral particles to fight against CaMV infection. On the other hand, virus-encoded P6 can interfere with the interaction between P4 and NBR1, thus allowing CaMV-induced viral inclusions and transmission bodies to antagonize NBR1 and therefore impeding the degradation of viral proteins and particles. This report revealed a potential strategy of viruses to evade autophagy degradation for successful infection (Hafrén et al., 2017). Autophagy also plays an antiviral role in barley stripe mosaic virus (BSMV) infection. However, BSMV suppresses autophagy via its encoded-γb protein, which directly interacts with ATG7 and competes for the ATG7–ATG8 interaction, which is essential for autophagy induction. As such, BSMV-encoded γb subverts autophagy-mediated antiviral defense responses by disrupting the ATG7–ATG8 interaction to facilitate infection (Yang et al., 2018) (Figure 1, lower part). More cases regarding the resolution of autophagy-mediated antiviral defense by plant viruses are reported hereafter. Further research is needed to unravel how viruses finely adjust to the induction and inhibition of the host autophagy pathway to achieve a perfect balance for their successful infection and colonization.

## Plant Virus Promotes Infection Through Host Autophagy

Apart from their direct interference with host plant autophagy, many viruses use autophagy to degrade some host plant factors, which have adverse effect on viruses, to promote their infection (Derrien et al., 2012; Cheng and Wang, 2017; Li et al., 2017) (Figure 1, lower part and Table 1).

In the course of plant–virus interactions, many viruses evolved mechanisms to manipulate host autophagy to meet their own needs. By degrading defense-related proteins through the autophagy pathway, plant viruses adversely affect plants to facilitate infection. Polerovirus encodes RSS P0 to trigger the degradation of the key RNA-silencing component ARGONAUTE1 (AGO1) via the autophagy pathway. Because AGO1 is co-localized with ATG8a in autophagic structures, the degradation of AGO1 was blocked following E64d or 3-MA inhibition treatment. Polerovirus-induced degradation of AGO1 via the autophagy pathway suppressed virus resistance and promoted viral infection in plants (Derrien et al., 2012). Similarly, TuMV infection caused the degradation of SGS3 and its preferential partner, RNA-dependent RNA polymerase 6 (RDR6). Treatment with the proteasome inhibitor MG132 or autophagy inhibitor 3-MA significantly attenuated virus-induced degradation of SGS3 and RDR6, suggesting that the ubiquitin proteasome and autophagy pathway are both involved in the degradation of important RNA-silencing components. TuMV-encoded VPg directly interacts with SGS3 and triggers SGS3 and RDR6 degradation, thereby attenuating host RNA-silencing and facilitating virus infection. Two other potyvirus-encoded VPgs, belonging to tobacco etch virus (TEV) and soybean mosaic virus (SMV), also interact with SGS3, indicating that the interaction between SGS3 and VPg and the degradation of the RNA-silencing component are general mechanisms of potyviruses (Cheng and Wang, 2017). The betasatellite of tomato yellow leaf China virus (TYLCCNV)-encoded βC1 up-regulates the expression of calmodulin-like protein (CaM), which interacts with the SGS3 protein in *N. benthamiana*. Transient co-expression of CaM and SGS3 induces autophagosomal activity to degrade SGS3 in host cells, while 3-MA treatment or silencing of *Beclin1, PI3K*, or *VPS15* in *N. benthasmiana* leading to inhibition of SGS3 degradation. TYLCCNV-induced CaM mediates RNA-silencing component SGS3 degradation, leading to successful infection of the virus in plants (Li et al., 2017).

Remorins are membrane-associated proteins found in *N. benthamiana* and rice plants that play crucial roles in cell-to-cell signaling and defense against biotic stress via the S-acylation (also known as palmitoylation) of C-terminal cysteine, a reversible post-translational modification contributing to cell plasma membrane (PM) localization and protein stability. Rice stripe virus (RSV) infection interferes with S-acylation of the C-terminal of remorin (NbREM1/OsREM1.4) through the viral movement protein NSvc4, which interacts with the NbREM1 C-terminal. NSvc4 competitively binds to the C-terminal of NbREM1/OsREM1.4 decreasing the S-acylated NbREM1/OsREM1.4 anchor to PM. S-acylation-deficient NbREM1/OsREM1.4 are sequestrated in the endoplasmic reticulum (ER) and induced autophagy for its degradation. Less S-acylated NbREM1/OsREM1.4 accumulation at the PM enhances the permeability of plasmodesma (Pd), thereby promoting virus infection (Fu et al., 2018). A study found that bamboo mosaic virus (BaMV) infection upregulated the expression of several *ATGs* and induced autophagy in *N. benthamiana* leaves. Autophagy inhibitor 3-MA treatment blocked autophagosome formation and reduced the accumulation of the viral coat protein, whereas rapamycin, an inducer treatment, enhanced the expression of the viral coat protein. BaMV-induced autophagy may offer an environment more conductive to viral replication or a shelter to evade from the RNA silencing (Huang et al., 2019). However, the autophagy mechanism contributing to BaMV RNA increase, and which viral protein of BaMV plays the crucial role in autophagy induction require further study.

## Conclusions and Future Directions

Autophagy plays a crucial role in the interaction between pathogens and mammalian cells (Shelly et al., 2009; Münz, 2017; Wang et al., 2018). In the past decade, an increasing number of studies expanded our understanding of autophagy in virus-attacked plants (Liu et al., 2005; Derrien et al., 2012; Hafrén et al., 2017; Haxim et al., 2017; Yang et al., 2018; Li et al., 2019). Most viruses trigger the autophagy pathway of host plants as a defense mechanism to counter virus infection. Notably, many viruses evolved to escape the autophagic machinery using distinct strategies, such as inhibiting autophagy induction, suppressing autophagosome nucleation, and blocking autophagosome fusion with vacuoles. Some viruses even hijack autophagic machinery for their replication and to increase movement within autophagosome-like vesicles. Therefore, accurate manipulation of plant autophagy has the potential to combat viral infections of plants in the agriculture field.

Both animal and plant viruses activate the autophagy pathway, which then induces viral protein and particle degradation as a defense mechanism (Shelly et al., 2009; Chan and Qu, 2017; Haxim et al., 2017; Sparrer and Gack, 2018; Li et al., 2019). The mechanism by which virus infection induces plant autophagy is still obscure, which viral proteins are key to inducing autophagy remains unknown, and the upstream signal of autophagy initiation upon virus infection is not yet clear. Understanding the induction of the autophagy mechanism in plants during virus infection will allow easier control of the autophagy pathway in the future.

Several ATGs act as cargo receptors or autophagy regulators to selectively interact with viral effectors, such as ATG8, which interacts with CLCuMuV βC1; ATG6, which interacts with TuMV NIb; and ATG7, which interacts with BSMV γb (Figure 1). These findings suggest that more autophagy-related proteins may interact with viral effectors and are involved in host immunity. Notably, βC1 binds to ATG8f, whereas Beclin1 binds to ATG8a but not ATG8f, and C1 binds to ATG8h but not to other ATG8s. This suggests that ATG8 has multiple homologs in plants to perform diverse functions. In addition, ATG8a binds to Beclin1 at the N-terminal AIM motif. NIb binds to Beclin1 at the C-terminal, and Beclin1-mediated degradation of NIb depends on ATG8a, indicating that Beclin1 acts as a bridge to guide viral proteins to autophagosomes for breakdown.

Furthermore, some ATGs are involved in other cellular signaling pathways, including cell death, cell–cycle regulation, and innate immune signaling (Wang, 2008; Xu et al., 2017; Levine and Kroemer, 2019). For instance, Beclin1, PI3K/VPS30, and ATG3 are all required to limit HR PCD to the pathogen infection site (Liu et al., 2005), ATG5 and ATG7 regulate glucose-induced ROS in *Arabidopsis* (Huang et al., 2018), and ATG9 is a negative regulator of innate immune signaling (Cadwell and Debnath, 2018; Levine and Kroemer, 2019). Investigating autophagy crosstalk with other cellular processes may provide researchers with new methods to modulate the autophagy pathway.

In the future, the molecular mechanisms and roles of autophagy during plant and virus interactions require further and deeper study. Genetic engineering approaches or chemical treatments can be harnessed to modulate autophagy to fight against plant viruses.

## Author Contributions

GZ and TZ designed the project. XH wrote the manuscript and reviewed the manuscript. SC, XiaY, XinY, and TZ edited the manuscript. All authors reviewed and approved the final version of the manuscript.

## Conflict of Interest

The authors declare that the research was conducted in the absence of any commercial or financial relationships that could be construed as a potential conflict of interest.
